# Dimensionality analysis of forearm muscle activation for myoelectric control in transradial amputees

**DOI:** 10.1371/journal.pone.0242921

**Published:** 2020-12-03

**Authors:** Alexander McClanahan, Matthew Moench, Qiushi Fu

**Affiliations:** 1 College of Medicine, University of Central Florida, Orlando, Florida, United States of America; 2 NeuroMechanical Systems Laboratory, Mechanical and Aerospace Engineering, University of Central Florida, Orlando, Florida, United States of America; 3 Biionix (Bionic Materials, Implants & Interfaces) Cluster, University of Central Florida, Orlando, Florida, United States of America; University of Illinois at Urbana-Champaign, UNITED STATES

## Abstract

Establishing a natural communication interface between the user and the terminal device is one of the central challenges of hand neuroprosthetics research. Surface electromyography (EMG) is the most common source of neural signals for interpreting a user’s intent in these interfaces. However, how the capacity of EMG generation is affected by various clinical parameters remains largely unknown. In this study, we examined the EMG activity of forearm muscles recorded from 11 transradially amputated subjects who performed a wide range of movements. EMG recordings from 40 able-bodied subjects were also analyzed to provide comparative benchmarks. By using non-negative matrix factorization, we extracted the synergistic EMG patterns for each subject to estimate the dimensionality of muscle control, under the framework of motor synergies. We found that amputees exhibited less than four synergies (with substantial variability related to the length of remaining limb and age), whereas able-bodied subjects commonly demonstrate five or more synergies. The results of this study provide novel insight into the muscle synergy framework and the design of natural myoelectric control interfaces.

## Introduction

It has been estimated that the 2005 upper limb amputation prevalence in the United States involved approximately 541,000 persons, and it was projected that the number of people living with a lost upper limb will double by 2050 [1]. Myoelectric control using electrical activity of forearm muscles (EMG) holds out significant promise as a natural interface between amputees and powered hand-wrist prostheses to restore manual dexterity and improve quality of life. However, despite decades of research, the usability of powered prostheses remains limited, and the rejection rates are still quite high [2]. While early methods such as direct control systems have provided the basis of active prosthetics, these approaches have been inadequate due to their limited function, limited movement fidelity, and occasionally unintuitive training [3, 4].

More recently, pattern recognition approaches have been studied extensively and implemented in commercial devices. These approaches detect muscle contraction patterns as discrete classes to drive function modules [5]. A variety of classification algorithms have been implemented and tested to discriminate muscle contraction patterns, such as support vector machines [6], random forest classifiers [7], linear discrimination analysis [8], and convolution neural networks [9]. Earlier pattern recognition approaches operate at fixed speed for each function module, whereas recent development has shown that augmenting the speed with the extraction of proportional ‘class activation’ information in addition to class labels could improve overall performance [10, 11]. While pattern classification accuracy and robustness have been improved over the years and two commercial systems have been developed, i.e., COAPT [12] and MyoPlus [13], the translation of these algorithms to clinical applications still proves challenging [3]. Pattern recognition algorithms inherently require long intervals to extract features for reliable classification. Moreover, since only one function module can be activated at a given time, sequential actions were often necessary to switch between function models for multi-joint tasks. Recent effort has been made to extract simultaneous actions (e.g., wrist motion plus finger motion) by defining more classes that represent functionally important motion combinations [14, 15]. However, this also indicates longer and more extensive training sessions to cover increased number of classes. Lastly, due to the discrete nature of the classifiers, incorrect classifications and unintuitive adaptation to changes in the interfaces (e.g., electrode shifts, arm position, etc.) could be challenging.

Considering the drawbacks of pattern recognition approaches, an alternative has been proposed to extract proportional control signals with multiple degrees of freedom (DoFs) simultaneously via linear [16–19] or nonlinear [20–22] regression algorithms. The promise of this approach is to allow flexible combinations of control signals from muscle contraction patterns associated with specific anatomical DoFs, thus leading to more intuitive myoelectric interfaces and allowing users to quickly adapt to small changes in the control mapping. This is possible because, according to the muscle synergy framework, multi-channel EMG signals contain information about supraspinal motor commands that activate synergistic muscle covariation determined by spinal cord circuits [23, 24]. A simultaneous and proportional myoelectric interface can therefore extract motor commands that are natural to the human user.

Though research into simultaneous and proportional control has demonstrated good performance in controlled environments as well as preliminary clinical use [25], there are still several important questions to be solved for clinical success. One of these questions is how muscle synergies are affected in amputees with different clinical conditions. The dimensionality of the muscle activation space (i.e., number of muscle synergies) is critically important for the reliability and usability of simultaneous and proportional control, since it determines *how many and which* DoF of the terminal devices can be driven by the extracted motor commands. However, due to the lack of systematic investigations into the muscle control capabilities of amputees featuring a large number of subjects, our understanding is very limited on this matter. Most clinical assessments studies with large sample size focused on the functional outcomes with time-based or subjective criteria [26–28], which cannot separate the contribution of myoelectric control and the terminal devices. In contrast, laboratory evaluation of myoelectric control often uses abstract tasks that are independent from terminal devices, but only pre-determined DoFs or classes was tested in able-bodied persons and a few amputee subjects. One of the few relevant works demonstrated that the ability to produce discrete forearm muscle contraction patterns (via pattern recognition classifiers) is correlated with residual forearm length, time since amputation, and phantom limb sensation, with a maximum of 11 independent patterns [29]. To the best of our knowledge, however, no research has quantified the muscle control capability of amputees within the framework of motor synergies.

The main goal of the present investigation is to quantify the dimensionality of synergistic muscle activation in a relatively large number of amputated subjects and to compare the results to able-bodied subjects. To this end, we have conducted a retrospective analysis of a publicly available dataset, NinaPro, featuring both able-bodied controls and transradially amputated subjects with different clinical parameters [30]. This dataset was established primarily to help the scientific community to evaluate movement recognition and force control algorithms for prosthetic hands. Since a large number of movement patterns of the hand and wrist were produced by the subjects, we were able to apply synergy extraction algorithms on this dataset to evaluate the heterogeneity in the dimensionality of muscle control space across individual patients with different characteristics.

## Materials and methods

### Source of data

The data used in this study was obtained from the NinaPro dataset [29]. This is a publicly available dataset the prosthesis research community uses to study the interplay between surface EMG (sEMG), hand kinematics/kinetics, and clinical parameters with the goal of movement classification for myoelectric control. 67 able-bodied subjects and 11 transradial amputees were enrolled in the study from which the *Ninapro* dataset was compiled. Subjects were tasked to perform various repetitive hand/wrist movements while having sEMG recordings taken from their forearm. Informed consent was obtained from each subject prior to the experiments, which was approved by the institutional review board. This dataset is divided into three subsets. Database 1 (27 able-bodied subjects) was excluded because it used different data collection protocol. We chose to only use Database 2 (40 able-bodied subjects) and Database 3 (11 transradial amputees) since they share the same movement exercises and electrode configurations across the two subject groups. A brief overview of the datasets is given below, and more details can be found elsewhere [29].

### Subject characteristics

Database 2 (**DB2**) contains data from 40 able-bodied subjects who had the following characteristics: 28 males, 12 females; 34 right-handed, 6 left-handed; and age 29.9 ± 3.9 years. Database 3 (**DB3**) contains data from 11 trans-radial amputees (all males; 10 right-handed, 1 left-handed; and age 42.36 ± 11.96 years). These amputees varied in their percentage of remaining forearm and this data was included in the *Ninapro* dataset along with other vital characteristics (years since amputation, amputated hand, DASH score, phantom sensation, and past experience with prosthesis; Table 1). Note that all amputees had acquired limb loss after adolescence, and there were no data from amputees with congenital limb loss. We note that one amputation was due to cancer, whereas others were caused by trauma. Radiotherapy have been reported to be an effective treatment for cancer in hand and foot, but it may cause local complications to joints and muscles adjacent to the targeted site [31]. However, given that this individual had a 90% residual arm length, it is reasonable to assume that radiotherapy (if any) was delivered to the hand, and it should not affect the function of forearm muscles. In fact, a recent study with breast cancer demonstrated that the alternation of musculature was observed in pectoralis muscles that are directly under treatment, but not in rectus abdominis muscles that are away from treatment [32].

**Table 1:** Summary of clinical characteristics of database 3.

Subject	Amputated hand	Cause of Amputation	% Forearm Remaining	Age	Years since Amputation	Phantom Limb Sensation (0–5)	DASH Score	Experience with prosthesis	Movements Analyzed	# of Electrodes Used
1	R	Accident	50	32	13	2	1.67	Myo	29	10
2	L	Accident	70	35	6	5	15.18	Cos	40	10
3	R	Accident	30	50	5	2	22.50	Myo	39	10
4	R&L	Accident	40	34	1	1	86.67	No	40	10
5	L	Accident	90	67	1	2	11.67	Kin	40	10
6	L	Accident	40	32	13	4	37.50	Kin	40	8
7	R	Accident	0	35	7	0	31.67	No	40	8
8	R	Accident	50	33	5	2	33.33	Myo	40	10
9	R	Accident	90	44	14	5	3.33	Myo	40	10
10	R	Accident	50	59	2	5	11.67	Myo	40	10
11	R	Cancer	90	45	5	4	12.50	Myo	33	10

DASH = disabilities of the arm, shoulder, and hand. Experience with prosthesis: Myo = myoelectric, Cos = cosmetic (passive), Kin = body powered, and No = no experience.

### Experimental protocol

Subjects in the *Ninapro* dataset underwent a series of four exercises (A-D) which consist of different hand movements or force patterns. Exercise A includes single finger movement but it was not performed in DB2 and DB3 that were selected in the current study. Exercise D focuses on isometric force exertion of one or more fingers, which were relatively difficult to perform by the amputees (only 8 of 11 completed Exercise D). Therefore, our study only used Exercises B and C (total 40 movement types, see Table 2). Specifically, Exercises B focused on movement of one or more joints without explicit functional relevance, whereas exercise C focused on functional joint coordination. For all movement tasks, subjects were shown a movie of the movement on a monitor: able-bodied subjects were asked to duplicate the movement with their right hand, while amputated subjects were asked to contract the muscles in their missing limb to mimic the movement as naturally as possible. Each movement was repeated six times, and the sequence of movements was not randomized with the objective to encourage a consistent movement pattern. Note that the dataset is missing the record of several movements for a few amputee subjects (Table 1, all missing data were from Exercise C).

**Table 2:** Summary of movements and force patterns in each exercise.

Exercise B		Exercise C	
Thumb up	13. Wrist flexion	1. Grasp around large diameter object	13. Tripod grasp
2. Extension of index and middle, flexion of all others	14. Wrist extension	2. Grasp around small diameter object	14. Prismatic pinch grasp
3. Flexion of ring and little finger, extension of all other	15. Wrist radial deviation	3. Fixed hook grasp	15. Tip pinch grasp
4. Thumb opposition toward base of little finger	16. Wrist ulnar deviation	4. Index finger extension grasp	16. Quadpod grasp
5. Abduction of all fingers	17. Wrist extension with closed hand	5. Medium wrap	17. Lateral grasp
6. Fingers flexed into a fist		6. Ring finger grasp	18. Parallel extension grasp
7. Pointing of index from fist		7. Prismatic four finger grasp	19. Extension type grasp
8. Adduction of extended fingers		8. Stick grasp	20. Power disk grasp
9. Wrist supination around axis of middle finger		9. Writing tripod grasp	21. Open a bottle with tripod grasp
10. Wrist pronation around axis of middle finger		10. Power sphere grasp	22. Turn a screwdriver while grasping with stick grasp
11. Wrist supination around axis of little finger		11. Three finger sphere grasp	23. Cut object (knife grasp with index finger extension grasp)
12. Wrist pronation around axis of little finger		12. Precision sphere grasp	

### Data acquisition and signal processing

The EMG recording setup was the same across 40 movements in Exercise B and C for each subject. For most subjects, a total of 12 sEMG electrodes (Trigno Wireless, Delsys, Inc) were placed on the subject’s arm at the following locations: (1) eight electrodes evenly spaced around the forearm at the radio-humeral joint; (2) two electrodes on the flexor digitorum superficialis (FDS) and the extensor digitorum superficialis (EDS); and (3) two electrodes on the biceps brachii (BB) and the triceps brachii (TB). sEMG signals were sampled at 2 kHz. There were two amputee subjects who only wore 10 electrodes (DB3 subjects 6 and 7 were missing electrodes at FDS and EDS). The sEMG signals were cleaned of 50 Hz power-line interference using a Hampel filter before being uploaded to the online data repositories. Note that the data collection of the Ninapro dataset combined both dense sampling approach (8 electrodes around forearm) and anatomical positioning strategy (electrodes on FDS, EDS, BB, TB) for electrode placement. The dense sampling approach (main signal source of the present study) does not target specific muscles. Additionally, the electrodes on FDS and EDS cannot precisely capture sub-group (i.e., finger specific) muscle activities. While this electrode placement is different from the common approach used in muscle synergy studies that target specific muscles, it aims to characterize the muscle activation pattern without a priori knowledge of muscle locations. This is practically important given differences in limb reduction among amputees, and has been the standard electrode placement for developing simultaneous and proportional myoelectric controllers [25, 33].

### Data analysis

After the data was obtained, we first used a fourth-order lowpass zero-lag Butterworth filter with a cutoff frequency of 3Hz on the rectified EMG signals. Then we down sampled the data to 100Hz, which was segmented using the movement label provided by the dataset. All signals from the ‘Rest’ periods were removed, whereas the remaining signals were concatenated across trials and normalized to have unit variance. The two sEMG channels on the BB and TB were not used in the analysis because they are not involved in hand/wrist movements. The quantification of the dimensionality of the EMG data across a broad range of movements are described below.

### Estimating the number of synergies

The non-negative matrix factorization (NMF) algorithm, a trusted method for analyzing high-dimensional data [34], was used to extract time-invariant muscle synergies and their time-dependent activation coefficients from the EMG data. Much of the following data analysis was derived from previous studies that extracted muscle synergies [35–37]. The ultimate goal of NMF is to capture major EMG channel co-variation patterns (i.e., synergies) within a pool of EMG samples obtained across many movements [38], thereby yielding an estimation of the dimensionality of the EMG data. The NMF can be described as:

E≅W*H (1)

where ***W*** is a n by k non-negative matrix representing k synergies for n electrodes and ***H*** is a k by T non-negative matrix representing the synergy activation coefficients for T samples. This decomposition was implemented using a common multiplicative update algorithm [39]. The accuracy of the reconstruction typically increases as a function of k. Various methods have been implemented to determine the minimum number of k that captures most of the total data variance [40–42], which are based on different methods that estimate the ‘variance accounted for’ (VAF) metric. In the present study, the VAF is defined as

$$VAF=100*\left(1-\frac{SSE}{SST}\right),$$ (2)

where SSE represents the sum of squared differences between the original and reconstructed EMG data, and SST represents the sum of the squared original EMG data. This definition was selected over the Pearson correlation coefficient due to its sensitivity to the magnitude of the data in addition to the shape [35]. The calculation of VAF was done both globally (global VAF) as well as within each electrode column (local VAF), such that the subtleties of the data at both levels can be captured.

To identify the number of synergies for each subject, we first divide the EMG dataset randomly into two subsets: extraction and validation, with 75% and 25% of total samples respectively. The NMF was first computed using the extraction subset with a given synergy number k, and the resulting synergy matrix W was used to obtain H using the validation subset. Then both global and local VAF after NMF were obtained from the reconstruction of the validation subset. Since the NMF algorithm may converge to local minima, the synergy extraction was repeated 50 times for each synergy number with random subset sampling and random initial estimates of ***W*** and ***H***. The result corresponding to the maximum global VAF for the given synergy number was selected. This entire procedure was performed with varying number of synergies k from 1 to n (i.e., number of electrodes). The number of synergies for each subject was defined as the minimum k that achieved a global VAF > 95% and a local VAF > 85% for each EMG channel.

For able-bodied subjects in DB2, we estimated the number of synergies using two electrode configurations. One consists of all forearm electrodes (n = 10), whereas the other consists of the eight evenly spaced forearm electrodes (n = 8). For the amputee subjects in DB3, the number of synergies was estimated either using the actual forearm electrode configuration (n = 8 for two subjects and n = 10 for the other nine subjects), or only 8-electrode configuration.

### Representative movements

Muscle synergies can normally be visualized and compared across subjects or between limbs if precise anatomical electrode locations are used. However, it was challenging to do this in the present study given the inconsistency of electrode locations due to the prosthesis-oriented electrode placement method used during data collection. Therefore, we took a different approach to qualitatively demonstrate the underlying neuromuscular structure of the extracted synergies. This approach was designed with the following assumptions: (1) the extent to which a given muscle synergy is activated varies across different movements, and (2) subjects share similar muscle synergies that are associated with movements of one or a set of joints (e.g., wrist extension or finger flexion). These assumptions can be justified by previous studies that examine muscle synergy in upper limbs [35, 43, 44]. Therefore, we defined the structure of the synergies by representing each extracted synergy in terms of activation level across movements. Specifically, for each synergy extracted from one subject, a 17 × 1 feature vector was defined. Each element of this vector is the averaged activation coefficient of this synergy for all repetitions of one movement type from Exercise B. The feature vectors of each synergy were normalized to zero-mean and unit standard deviation to remove the scaling ambiguity of the NMF. In other words, the feature vectors describe the relative activation strength of a given synergy across all simple movements. We excluded Exercise C movements when building feature vectors because Exercise C includes mostly functional grasping and manipulation movements, which are more complex and challenging to perform consistently due to inter-personal preferences than joint-based Exercise B movements. This between-subject variability would significantly deteriorate the reliability of the clustering result (i.e., less average Silhouette score, see below).

Representative movements that were strongly associated with synergies (i.e., high activation coefficients) were revealed using clustering analysis on these feature vectors pooled from all able-bodied subjects. Specifically, we used k-means clustering method with 1-*r* as distance metric, in which *r* is the Pearson’s correlation coefficients between two feature vectors. We repeat k-means algorithm 50 times for a given k (from 2 to 15), and the optimal number of clusters was determined using the Silhouette scores averaged across all samples. The Silhouette score is a metric that quantifies how similar a sample is to its own cluster compared to other clusters [45]. A higher Silhouette scores represents better clustering quality. Subsequently, we defined the primary representative movements as those with the strongest synergy activation, which are all at least 2 S.D. greater than the mean activation strength within each cluster. Additionally, secondary representative movements were also defined in some clusters if the movement with the second strongest activation was 1 S.D. greater than the mean.

Lastly, we compared synergies extracted from amputee subjects with those extracted from able-bodied subjects. We choose not to cluster synergies from amputees because the high degree of heterogeneity that make the clustering less reliable (i.e., low Silhouette scores). Instead, we tried to assign these synergies to the closest synergy cluster extracted from the able-bodied group. This was accomplished by computing the distance metric (i.e., 1-*r*) between a given amputee synergy vector and the centroid of an able-bodied cluster. The amputee synergy is considered similar to a cluster with the smallest distance, if this distance is smaller than the maximum within-cluster sample-to-centroid distance. Note that we only performed the analysis described in this section using synergies extracted from 10-electrode configuration. Two amputee subjects with 8-electrode configuration were not examined because it was found that they had very low number of synergies (see **Results**).

### Statistical analysis

We seek to examine the effect of clinical parameters on the number of synergies in amputee subjects (DB3). Spearman’s correlation coefficients and the corresponding significance were obtained using number of synergies as the dependent variable and residual arm length, years after amputation, age, and phantom sensation as independent variables. The DASH score was not used because it correlates with the residual arm length and is influenced by many factors, e.g., type of prosthesis use and side(s) of amputation. We used t-tests for comparing subject age, height and weights. Moreover, we used nonparametric statistical tests (Wilcoxon Signed Ranks Test and Mann-Whitney U Test) for comparing the number of synergies between groups or conditions, as well as other subject characteristics.

## Results

### Dimensionality of forearm surface EMG in able-bodied subjects

**Fig 1:**
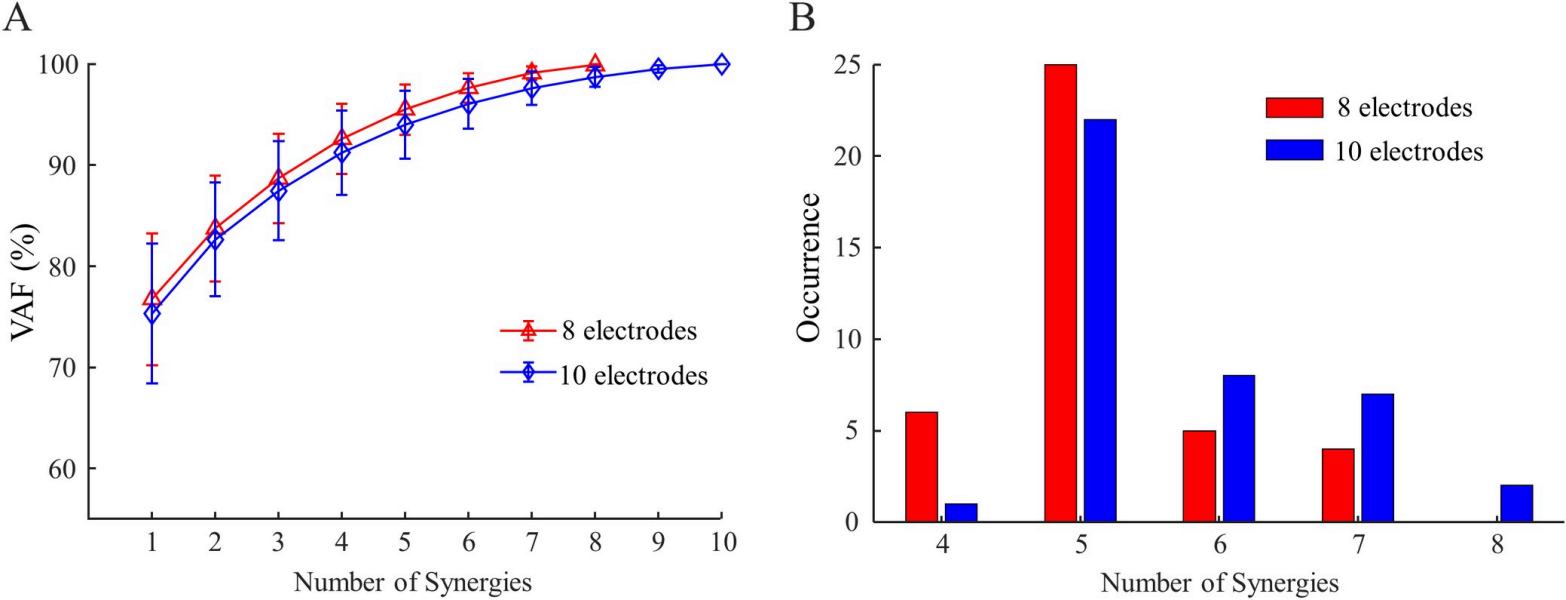
Dimensionality estimation of forearm muscle activation in able-bodied subjects. (A) EMG data variance explained (VAF) by synergies extracted from able-bodied subjects, plotted as electrode count increased. (B) Histogram distribution of the number of synergies extracted from all forty able-bodied subjects.

The dimensionality of forearm muscle EMG across 40 movements were estimated in able-bodied adults using either an eight-electrode configuration or a ten-electrode configuration. As the number of synergies used to reconstruct the raw data increased, more variance was explained by the NMF algorithm in both configurations (Fig 1A). To meet our criteria on both global and local VAF, it was found that five synergies were required in most subjects (Fig 1B), with only a few subjects showing less than five synergies. The global VAF of five synergies reconstruction were 95.5 ± 2.5% and 94.0 ± 3.4% (mean ± SD) for eight- and ten-electrode configurations respectively. The addition of two electrodes located at the extrinsic finger muscles led to a significantly larger estimation of the dimensionality (p < 0.001). Specifically, the use of ten-electrode configuration yielded 5.7 ± 1.0 synergies, whereas the eight-electrode configuration yielded 5.2 ± 0.8 synergies (reduction in 17 of 40 subjects). Subject height, weight, and age were found not to predict the number of synergies. However, we found male subjects exhibited a larger number of synergies than female subjects in both eight-electrode (5.3 ± 0.8 versus 4.8 ± 0.9) and ten-electrode (5.9 ± 0.9 versus 5.2 ± 1.0) configurations. Both differences were significant (p = 0.039 and 0.015, respectively). Considering the significant difference in body sizes (i.e., height and weight, both p < 0.001) between two genders, we think that this difference may be linked to the volume conduction in forearms with different sizes (see discussion).

### Dimensionality of forearm surface EMG in amputee subjects

The amputee subjects were older than the male control subjects (42 ± 12 versus 30 ± 4 years; t-test, p < 0.001), but they had similar height and weight. The EMG dimensionality in amputee subjects was first estimated with the actual electrode configurations (9 had ten electrodes, 2 had eight electrodes). With a global VAF of NMF reconstructions of 96.2 ± 0.5%, it was found that the number of synergies extracted from these subjects was highly variable: between 1 and 7, averaging less than four (3.6 ± 1.7). As expected, this is significantly less than the dimensionality estimated from able-bodied subjects (p < 0.001 compared to both eight- and ten-electrode configurations). This difference remains (3.9 ± 1.5 synergies) if we exclude subject 7 as an outlier due to his significant loss of muscle volume (~0% residual limb length) compared to other amputee subjects. We also estimated the EMG dimensionality with only eight evenly spaced electrodes, but the result was not statistically different from the one obtained with all available electrodes. A close examination of the results showed that the number of synergies decreased by one for the four patients with ≥ 70% residual arm length, but it did not change for other patients. This means that FDS and EDS electrodes had little independent contribution to the EMG dimensionality in trans-radial amputees with medium to high level of amputation.

**Fig 2:**
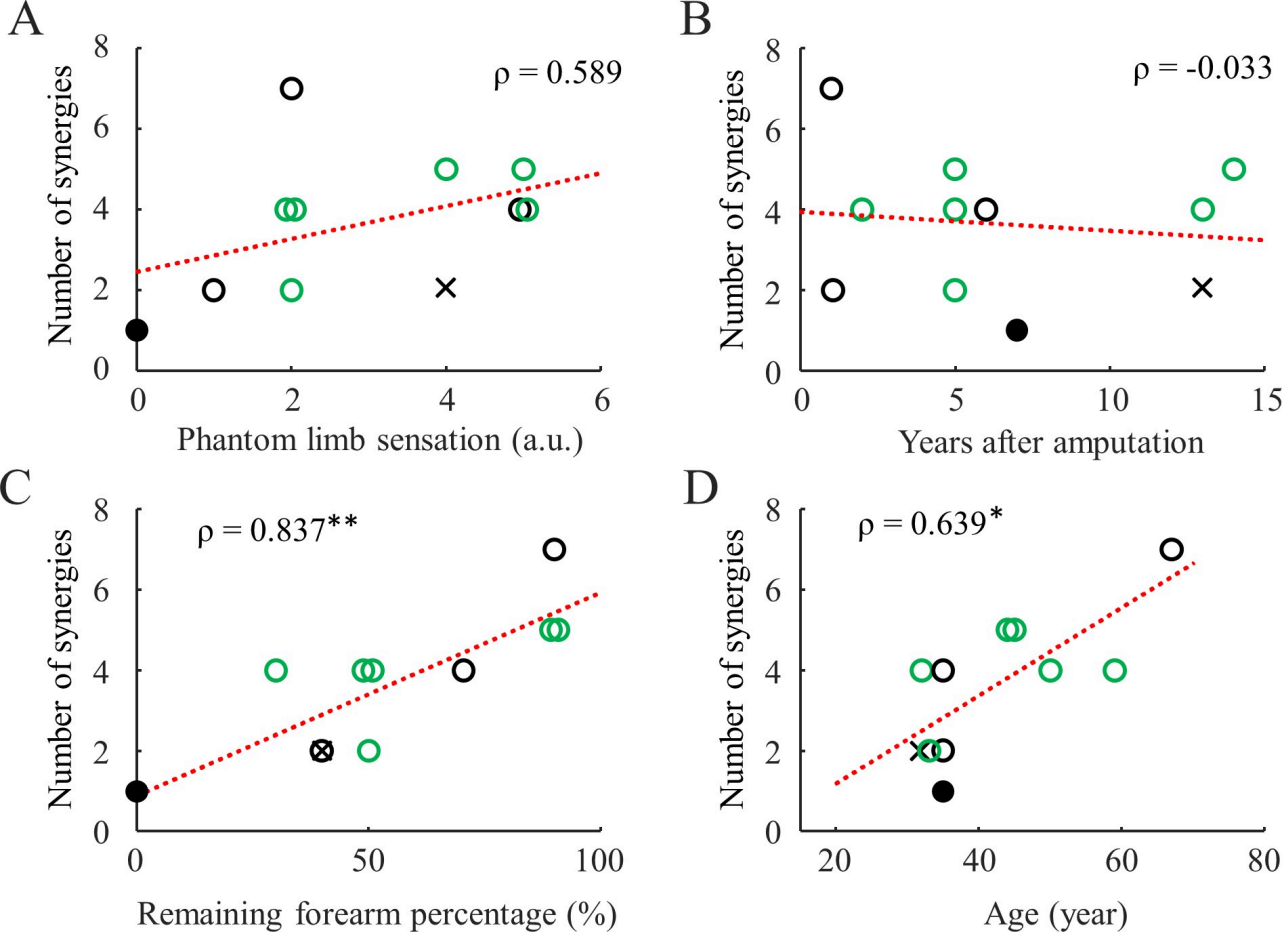
Relation between clinical parameters and number of synergies in amputee subjects (DB3). The red dashed lines are the linear fit lines for all subjects. Spearman’s coefficient is shown on the side. The filled circle and cross represent Sub7 (zero remaining forearm) and Sub6 (8 electrodes), respectively. The green circles represent myoelectric device users. Single asterisk and double asterisks indicate *p* < 0.05, and *p* < 0.01 respectively.

With all available electrodes, we found that the number of synergies cannot be predicted by either phantom limb sensation intensity (Fig 2A) or time since amputation (Fig 2A). In contrast, the residual limb length and age were both found to be positively correlated with the number of synergies (Fig 2C and 2D). These statistical significance did not change if we removed subject 7 as an outlier. Lastly, we did not find differences in the number of synergies between myoelectric device users and non-users who had the same average residual limb length.

### Representative movements for extracted synergies

**Fig 3:**
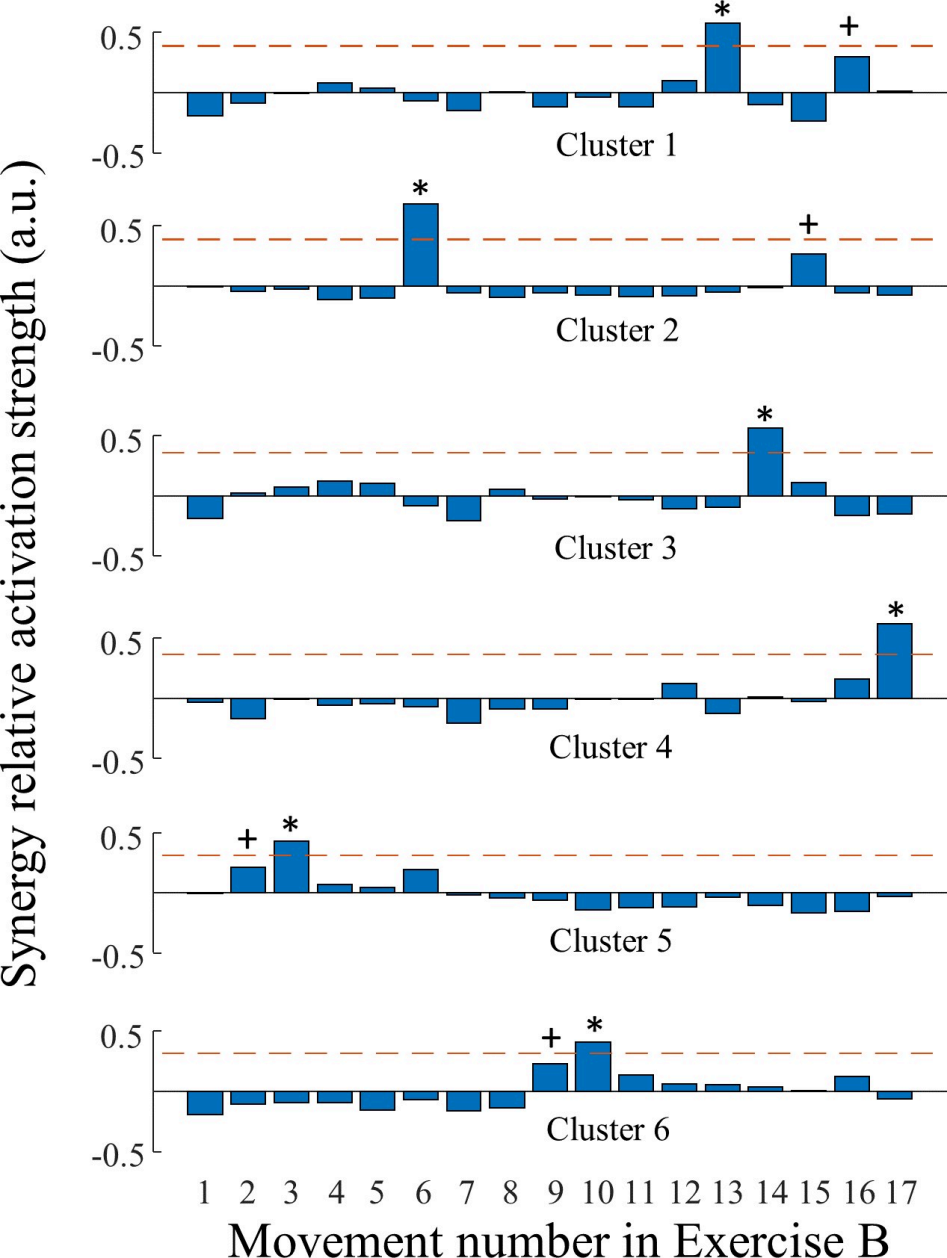
Activation strength of synergy types extracted from able-bodied subjects. Each row represents the relative activation strength for movements from Exercise B, averaged within a cluster of normalized synergies. The dashed horizontal lines represent the Mean + 2 S.D. of these averaged activation strength. The asterisks and crosses indicate the primary and secondary representative movements for the synergy clusters. The movement number is defined in Table 2 Exercise B.

To understand the neuromuscular structure of the extracted synergies, we first pooled all feature vectors corresponding to each synergy extracted from all able-bodied subjects (only for the 10-electrode configuration). As described earlier, each feature vector represents the relative activation strength of a given synergy across 17 movements performed by one individual. There was a total of 227 feature vectors, which were analyzed using k-means clustering with silhouette scores to reveal an optimal cluster number of six. This indicates that there were *at least* six distinct types of muscle synergies extracted from able-bodied subjects. Fig 3 illustrates these synergy types represented by the centroids of these clusters (in terms of relative activation strength across movements). These synergies can be qualitatively described using representative movements that had significantly larger activation strengths (Table 3).

**Table 3:** Characteristics of clusters computed from the synergy pool of 40 able-bodied subjects.

Cluster No.	No. of Synergy samples	% variance explained in EMG per synergy	Primary (and Secondary) representative movements
1	53	15.8 ± 6.0	Wrist flexion (Wrist ulnar deviation)
2	45	16.7 ± 5.5	Finger flexion (wrist radial deviation)
3	42	23.0 ± 7.8	Wrist extension
4	36	13.9 ± 5.2	Wrist extension & finger flexion
5	27	14.1 ± 4.7	I&M finger extension (R&L finger flexion)
6	24	15.2 ± 6.7	Wrist pronation (Wrist supination)

The structure of the synergies extracted from 9 of 11 trans-radial amputees, who had 10 electrodes, was examined with respect to these synergy clusters formed within able-bodied subjects (Table 4). We tried to define the type of each synergy using a cluster from able-bodied subjects. However, 5 out 37 synergy samples cannot be defined because their distances to any cluster centroid are too far. For the remaining 32, the most common type is Cluster 2 (8 subjects), whereas the least common type was Cluster 5 (1 subjects). The appearance of other cluster types is quite variable (Cluster 1: 6 subjects, Cluster 6: 4 subjects, Cluster 3: 4 subjects, Cluster 4: 3 subjects).

**Table 4:** Classification of synergy type in amputee subjects with respect to the synergy clusters of able-bodied subjects.

Subject (% arm)	Identified synergy (cluster) type ranked by variance explained						
1 (50)	**1** (31.8)	**6** (30)	**6** (18.1)	**2** (15.0)			
2 (70)	**6** (27.6)	**2** (24.5)	**1** (21.8)	**6** (21.1)			
3 (30)	**3** (44.3)	**2** (35.7)	**4** (10.9)	**0** (4.1)			
4 (40)	**2** (48.4)	**5** (46.6)					
5 (90)	**4** (24.6)	**6** (22.5)	**0** (16.7)	**0** (14.8)	**1** (6.9)	**0** (5.5)	**1** (4.0)
8 (50)	**1** (51.3)	**2** (43.7)					
9 (90)	**0** (27.1)	**1** (22.0)	**2** (17.1)	**3** (17.0)	**4** (11.8)		
10 (50)	**1** (32.1)	**3** (26.0)	**6** (19.8)	**2** (17.1)			
11 (90)	**3** (25.1)	**3** (24.3)	**2** (19.0)	**2** (14.0)	**6** (12.6)		

Sub6 and Sub7 were excluded. The cluster number refers to Fig 3 and Table 3, and zero indicates no appropriate cluster was found. These synergies are ranked with a descending value of explained variance (the numbers in the parentheses following the cluster number) in the corresponding subject’s EMG.

## Discussion

Our results demonstrated that the synergistic activities of forearm muscle contractions are generally impaired in trans-radial amputees, and the extent to which the dimensionality of the muscle contraction is reduced is related to several clinical parameters (level of amputation and age). Importantly, we identified six main synergy types that are common in able-bodied individuals, and we classified amputee synergies with these types. It was found that, although synergies representing wrist and finger flexions were mostly preserved, there were high degrees of heterogeneity in the residual muscle synergy structure across amputees. We discuss our results with respect to the muscle synergy framework and prosthesis control applications below.

### Methodological limitations

The present study is a retrospective analysis using an open-source dataset. While the dataset contains a wide range of movements and a relatively large number of control and amputee subjects, there are two main limitations associated with the present study and the results. First, the EMG signals were obtained from surface locations that are not precisely linked to individual functional muscles. This prevents physiologically accurate extraction and direct comparison of muscle synergies, due to spatial uncertainties associated with the electrode locations. Therefore, this study only provides an approximation of the muscle synergies and their dimensionality. Ideally, the EMG signals for synergy extraction should be acquired at precise anatomical sites [18, 43, 44], or using high-density electrode arrays that provide more spatial information [38, 46]. Moreover, EMG recordings from the intact limb should be acquired to enable within-subject comparisons. Second, the movements included in the original dataset are missing in some cases that could potentially improve the estimation of the dimensionality of forearm muscles, a notable one being the extension of all fingers. Also, the dataset includes only a few movements that focus on coordinated motion of the fingers and wrist, which are functionally important for manual dexterity [47]. Future studies that quantify muscle control capabilities in amputees should consider these limitations and adjust the experimental protocol accordingly. Despite the inaccuracies in estimating the exact physiological structure of the muscle synergies, our results obtained from the able-bodied control group is consistent with previous studies, suggesting the validity of the present method. We believe that the present study provides good estimations about the dimensionality of the forearm muscles contractions, which has several important indications to the clinical investigation of non-invasive myoelectric control.

### Dimensionality of hand/wrist muscle contraction in able-bodied individuals

The muscle synergy framework has emerged in recent years as a technique to understand how motor control is coordinated across combinations of muscles, with applications for clinical assessment and rehabilitation after injuries and diseases that impair the neural pathways of sensorimotor control [48, 49]. It has been argued that multiple muscles can generate covarying activities as a ‘functional unit’, i.e., muscle synergy, which enables the central nervous system to operate in a low-dimensional functional (neural) space instead of a high-dimensional muscle/joint (mechanical) space for common tasks [24, 50]. Such dimensionality reduction can often be quantified using matrix factorization methods such as principle component analysis (PCA), independent component analysis, and NMF on EMG signals [51]. Precise estimation of the dimensionality of hand/wrist muscle activation is challenging because experimental setups do not usually record from all relevant muscles given the many DoFs and complex musculoskeletal structure of the forearm. Weiss and Flanders demonstrated approximately 3–4 synergies were needed to account for > 90% of the variance of EMG recorded from five intrinsic and two extrinsic finger muscles during grasping or spelling tasks [44]. Manickaraj et al., identified 2–3 synergies from five forearm muscles to account for > 90% of the variance during wrist movement tasks [52]. Zariffa et al., showed 5 synergies can be extracted from eight electrodes (> 85% variance explained) across in-hand and forearm muscles during functional grasping tasks [43]. The structure of the synergies varies greatly across these studies due to the differences in electrode configuration and measured movements. Therefore, these investigations only captured a subset of the hand/wrist motor synergies, considering that kinematic analysis indicates 9 synergies were needed to explain > 90% variance measured across 19 finger joints during activities of daily living [53]. Constrained by the experimental setup of the dataset used in the current study, we demonstrated approximately five to six synergies for each able-bodied individual which can be clustered into six distinct types (Fig 3). The variation of the individual synergy number could be caused by subject-to-subject variations in muscular structure and electrode placement. One can observe that these extracted synergy types do not explain most of the finger movements. This was expected since the electrode configuration does not include intrinsic finger muscles, and it only grossly focused on wrist and extrinsic finger muscles, which may vary little across finger movements. An important finding is the effect of gender: less synergies were extracted from female subjects than from male subjects. Gender was mostly overlooked in previous studies of upper-limb muscle synergies due to small sample sizes. For the present study, we think that this effect could be best explained by body size differences. It was found that forearm circumference of females is about 16% less than that of males [54]. A larger circumference of the forearm enables larger inter-electrode distances, which allows the sensors to capture more independent muscle activities. Future studies are needed to better examine this finding.

### Dimensionality of hand/wrist muscle contraction in transradial amputees and the effects of clinical parameters

The structure and activation of muscle synergies of upper limbs can be altered by impaired sensorimotor neural pathways, such as those found in stroke [37], spinal cord injury [43], dystonia [55], and pain [52]. Most of these previous studies focused on injury/diseases occurring at or above spinal motor neuron level. In contrast, amputation could lead to several distinct damage to the motor system at peripheral sites. First, muscular structure can be significantly altered due to surgical management. Specifically, for transradial amputation, myodesis of deeper forearm muscles and myoplasty of superficial muscles are needed for bone coverage and contraction stability post-surgery [56]. These procedures, as well as retractions and fibrosis after surgery, may alter the conduction of the muscle unit action potentials within the forearm tissue due to changes of the source signal locations and tissue conductivity. Consequently, the pattern of surface EMG signals could be altered in an even-spaced electrode setup as in most myoelectric control applications. Such disturbance to the musculature could be less for individuals who have longer residual arm length [57, 58]. For instance, a distal third forearm amputation could leave the origin and insertion of the pronator teres and supinator intact, and tenodesis can be used for more distal amputations in which tendons are preserved. Another factor that could potentially change volume conduction is the circumference of the forearm, as demonstrated in able-bodied subjects between genders. Although the amputees in this study have similar body size as the male able-bodied controls, surgery and muscle atrophy could cause reduction in the forearm circumference. However, we think this effect does not play relatively small role since only small difference (< 0.5 synergy) was found between males and females. This is also supported by the fact that no difference was found between myoelectric device users and non-users, considering the latter are more likely to develop atrophy due to non-use. In addition to these changes at musculature level, changes in muscle synergies could also be attributed to the missing afferent signals. Although questions remain regarding the contribution of sensory feedback in organizing and activating muscle synergies, it has been demonstrated that deafferented frogs exhibit different synergy structure and activation compared to intact frogs. Moreover, it was demonstrated that deafferentation could induce phantom limb pain and reorganization of cortical somatotopic map [59, 60], which may led to alternation of synergy structures as seen in Table 4. Therefore, it is possible that the lack (or alteration) of sensory feedback from the missing part of the limb could impact how synergies are modulated in amputee subjects (Table 3). Considering these two types of damage, our result that the dimensionality and the structure of forearm muscle contractions change as remaining limb length reduces can be expected.

We found that the dimensionality of forearm muscle contraction does not correlate with the number of years after amputation (Fig 3B). This could suggest that natural usage (contraction) of the muscles may not be important to maintain muscle synergies as those synergies are already well developed in these patients before trauma induced amputation. In contrast, we did find that the number of synergies increase as a function of age (Fig 3D). However, it is difficult to speculate why this was the case because age did not strongly predict the number of synergies in able-bodied subjects, and the sample size of amputee subjects is considerably smaller than the able-bodied subject dataset. This correlation could be an artifact caused by other unreported clinical parameters mentioned in the previous paragraph.

Lastly, we would like to compare the present study to the work of Atzori and colleagues [29], in which the same dataset was used to examine muscle control in the form of generating discrete muscle activation patterns. These independent patterns were identified by using classification algorithms on the EMG data for each amputee subject, and the number of discrete patterns was determined as the largest subsets of 40 movements (Exercise B and C) that can achieve >90% classification accuracy. Therefore, this study quantified individual’s muscle contraction space as discrete movement classes. In contrast, our analysis quantified the muscle contraction space as axes on which the activations can co-vary continuously. Atzori et. al. demonstrated that the number of independent movements can vary between 2 and 11 within the amputee subjects (DB3), which is potentially smaller than that in able-bodied subjects [61]. This number is generally larger than the number of synergies we found in the current study, because each synergy can afford more than one independent movement at distinct activation levels. Furthermore, Atzori et. al. found that the number of independent movements can only be weakly predicted by the remaining limb length, but that they can be strongly predicted by the phantom limb sensation and number of years after amputation. A closer examination of the dataset suggested that six myoelectric prosthesis users had larger averaged number of years after amputation is (7.3 years) and higher averaged degree of phantom sensation (3.3) than five non-myoelectric users (5.6 years and 2.4, respectively) in this dataset (same trend can be observed if Sub 7 is excluded). Therefore, we speculate that the ability to produce discrete movements patterns can be improved by the experience of using prosthesis devices. Active use of myoelectric prosthesis requires muscle contraction on a day-to-day basis, which could lead to motor learning at the cortical level [62] that may help the patient to generate contraction patterns more consistently for a given movement and more distinct across movement, i.e., higher signal-to-noise ratio in offline classifiers. Indeed, it has been found that movement classification accuracy in pattern recognition based controllers was higher in myoelectric hand users than non-myoelectric users, and non-myoelectric users could get better if training is given [63]. Similar observation was also reported with functional clinical outcome measures for conventional myoelectric hands users (and non-users) [26]. Moreover, prosthesis use has been shown to maintain phantom sensation vividness better than non-use [64], and prosthesis use can preserve mental rotation ability [65] which is shown to be influenced by phantom sensation [66]. Lastly, neuroimaging studies have shown that use of myoelectric prosthesis could reduce cortical reorganization, i.e., the ‘invasion’ by neighboring cortical areas that may be related to the phantom limb pain [62]. Given the above evidence, we think that the ability to produce discrete muscle pattern involves both supraspinal structures which can be facilitated by usage of myoelectric prosthesis. In contrast, our current study addresses the muscle control capability mainly defined by the dimensionality of the overall EMG signal variance, which is less affected by the cortical level use-dependent changes.

### Clinical implications in neuroprothetics

NMF is one of the common methods to define simultaneous and proportional myoelectric interfaces in hand-wrist prosthesis, while other methods (e.g., regression, PCA) are also based on the muscle synergy framework to extract intuitive motor commands [67]. Although these methods are usually not completely unsupervised as implemented in the present study, the number of DoFs that can be controlled by such interfaces is still directly related to the dimensionality of the forearm EMG. Most existing studies that involve transradial amputees use 2-DoF interfaces, in which the first DoF is usually wrist flexion/extension [20, 68, 69]. This accords well with our result that a muscle synergy associated with this DoF is mostly intact in amputees with varying clinical parameters. The mapping of the second DoF in these studies includes: wrist pronation/supination [68], finger flexion/extension [69], and wrist radial/ulnar deviation [20]. These DoFs were all consistently found in the able-bodied subjects, but highly variable in amputees. In fact, some of the amputee synergies does not resemble any of the able-bodied synergies. Therefore, our results indicate that the optimal choice of 2-DoF interface is highly user dependent, and we cannot simply use one-size-fit-all approach in clinical testing. A suboptimal selection of DoFs could force the user to use synergies that have relatively small variances, which may limit the performance or increase the energy expenditure. 3-DoF interfaces have also been tested in able-bodied subjects [18, 38]. However, it may be challenging to define 3-DoF interfaces using surface EMG for amputees with remaining forearm length <70% due to the reduced muscle contraction space. Furthermore, motor synergies that involve partial hand motions may not be a good option for amputees, although it has been successfully implemented to drive a multi-DoF hand for able-bodied subjects [70]. In summary, clinical parameters play an important role in determining the DoFs for clinical implementations of simultaneous and proportional myoelectric interfaces, and studies with able-bodied subjects may not always be translational for clinical use. It is important to test patient’s muscle control capacity before fitting the terminal device for better customization (i.e., precision medicine). We propose that mechanical designs (e.g., how to map the DoFs) should also be customized to match the available dimensionality of the patient’s muscle control. Moreover, mechanically complimentary prosthetic hands could enhance the capability of simultaneous and proportional interfaces (limited by number of muscle synergies) by providing additional flexibility in day-to-day operations such as grasping [71, 72].

## Conclusions

Due to difficulties in recruiting subjects with upper limb amputation at single study sites, it is traditionally challenging to examine the characteristics of this population with a reasonable sample size. Most existing studies have used able-bodied subjects, and some have been able to test 2–3 amputee subjects. The present study analyzed the largest publicly available dataset of surface EMG recordings of hand/wrist movements performed by transradial amputees and able-bodied controls. We found that the dimensionality of muscle contraction in the forearm varies greatly in amputees, with correlation to the level of amputation and age. Building on these results, in future studies we will investigate how to personalize terminal devices for individuals with distinct muscle control capabilities to maximize usability, as well as examine the development of muscle synergies in patients with congenital limb amputation.
